# Expanding the therapeutic potential of *Salvia miltiorrhiza*: a review of its pharmacological applications in musculoskeletal diseases

**DOI:** 10.3389/fphar.2023.1276038

**Published:** 2023-12-05

**Authors:** Zhiqiang Ye, Yuyu Liu, Jintong Song, Yin Gao, Haiping Fang, Zilong Hu, Min Zhang, Wenwei Liao, Liao Cui, Yanzhi Liu

**Affiliations:** ^1^ Zhanjiang Key Laboratory of Orthopaedic Technology and Trauma Treatment, Zhanjiang Central Hospital, Guangdong Medical University, Zhanjiang, China; ^2^ Guangdong Provincial Key Laboratory for Research and Development of Natural Drug, School of Pharmacy, Guangdong Medical University, Zhanjiang, China; ^3^ Key Laboratory of Traditional Chinese Medicine for the Prevention and Treatment of Infectious Diseases, Guangdong Provincial Administration of Traditional Chinese Medicine (Central People’s Hospital of Zhanjiang), Zhanjiang, China; ^4^ Marine Medical Research Institute of Zhanjiang, Zhanjiang, China

**Keywords:** bone, joint, osteoarthritis, osteoporosis, *Salvia miltiorrhiza*, musculoskeletal diseases

## Abstract

*Salvia miltiorrhiz,* commonly known as “Danshen” in Chinese medicine, has longstanding history of application in cardiovascular and cerebrovascular diseases. Renowned for its diverse therapeutic properties, including promoting blood circulation, removing blood stasis, calming the mind, tonifying the blood, and benefiting the “Qi”, recent studies have revealed its significant positive effects on bone metabolism. This potential has garnered attention for its promising role in treating musculoskeletal disorders. Consequently, there is a high anticipation for a comprehensive review of the potential of *Salvia miltiorrhiza* in the treatment of various musculoskeletal diseases, effectively introducing an established traditional Chinese medicine into a burgeoning field. Aim of the review: Musculoskeletal diseases (MSDs) present significant challenges to healthcare systems worldwide. Previous studies have demonstrated the high efficacy and prospects of *Salvia miltiorrhiza* and its active ingredients for treatment of MSDs. This review aims to illuminate the newfound applications of *Salvia miltiorrhiza* and its active ingredients in the treatment of various MSDs, effectively bridging the gap between an established medicine and an emerging field. Methods: In this review, previous studies related to *Salvia miltiorrhiza* and its active ingredients on the treatment of MSD were collected, the specific active ingredients of *Salvia miltiorrhiza* were summarized, the effects of *Salvia miltiorrhiza* and its active ingredients for the treatment of MSDs, as well as their potential molecular mechanisms were reviewed and discussed. Results: Based on previous publications, Salvianolic acid A, salvianolic acid B, tanshinone IIA are the representative active ingredients of *Salvia miltiorrhiza*. Their application has shown significant beneficial outcomes in osteoporosis, fractures, and arthritis. *Salvia miltiorrhiza* and its active ingredients protect against MSDs by regulating different signaling pathways, including ROS, Wnt, MAPK, and NF-κB signaling. Conclusion: *Salvia miltiorrhiza* and its active ingredients demonstrate promising potential for bone diseases and have been explored across a wide variety of MSDs. Further exploration of *Salvia miltiorrhiza*’s pharmacological applications in MSDs holds great promise for advancing therapeutic interventions and improving the lives of patients suffering from these diseases.

## 1 Introduction

Musculoskeletal diseases (MSDs) encompass a wide range of disorders that includes muscles, bones, joints, tendons, and ligaments. MSDs take various forms that affect the human locomotor system. Common representative diseases include arthritis (osteoarthritis, OA; rheumatoid arthritis, RA), osteoporosis, fractures, sarcopenia, and tendonitis (World Health Organization, 2022). They particularly represent a range of conditions characterized by aging inflammation, degradation, and destruction of connective tissue in various sites of the locomotor system (Adami et al., 2019; Kaneko et al., 2022). These diseases not only cause chronic pain and limited mobility and flexibility for individuals, but also induce high disability and even mortality (Safiri et al., 2021). The direct costs associated with medical treatments, rehabilitation, and long-term care for individuals with MSDs are substantial. Moreover, there are indirect costs related to lost productivity and absenteeism from work (Blyth et al., 2019). The burden due to MSDs is growing worldwide along with the increasing aging population. Some musculoskeletal disorders, like osteoporosis (Langdahl, 2021) and rheumatoid arthritis (Fraenkel et al., 2021), have seen the development of several effective medicines to manage disease progression. For instance, in the case of osteoporosis, a range of medications has been developed, each with its own specific mode of operation. These include anti-resorptive agents (which inhibit osteoclast activity, such as Bisphosphonates and Denosumab), bone-forming agents (which stimulate osteoblast activity, such as Teriparatide), and dual-acting agents (which simultaneously stimulate osteoblasts and inhibit osteoclasts, such as Romosozumab). However, it is essential to recognize that most of MSDs are chronic and degenerative in nature. This implies that achieving complete recovery or a definitive cure may not be possible with the current treatment modalities (Conigliaro et al., 2019). Moving forward, it is imperative to continue research efforts aimed at developing novel therapeutic approaches. Early intervention and preventive strategies can play a pivotal role in mitigating the impact of MSDs on individuals’ quality of life (Williams et al., 2018; Cieza et al., 2021).

*Salvia miltiorrhiza* (SM) is a perennial herb belonging to the labiatae family, and its dried roots and rhizomes are commonly referred to as Danshen, regarded as “top-grade” herb in China. Its use dates back over 2000 years and was first recorded in the ancient Chinese pharmacological monograph <Shennong Ben Cao Jing>. SM is well-known for its diverse therapeutic properties, including promoting blood circulation, removing blood stasis (Wei et al., 2023), calming the mind, tonifying the blood, and enhancing the “Qi.” Over the centuries, the utilization of SM has exhibited remarkable efficacy in addressing a wide spectrum of ailments, including cardiovascular, neurological, cancer, osteoporosis, liver, gynecological, and chronic kidney diseases. It is utilized either as a standalone therapy or in combination with other traditional Chinese medicines. For instance, the combined administration of SM and astragalus membranaceus has been shown to ameliorate cyclosporin A-induced chronic nephrotoxicity through modulation of the gut-kidney axis. Danshen Zexie Decoction has exhibited effectiveness against non-alcoholic fatty liver disease by inhibiting the ROS/NLRP3/IL-1β pathway; Furthermore the combination of Danshen and gegen decoction has demonstrated a protective effect on hearts and cardiomyocytes in the model of post-ischemia reperfusion injury (Hu et al., 2012; Han et al., 2021; Biao et al., 2022).

Currently, most chemical ingredients of SM have been identified. Its main active ingredients consist of over 30 lipophilic compounds with diterpene groups and more than 50 hydrophilic compounds with phenolic acid groups. The lipophilic ingredients include tanshinone I-VI, cryptotanshinone, Isotanshinone I-II, tanshinol A, hydroxytanshinone, methyltanshinonate, and methylinitanshingwinoone. On the other hand, the hydrophilic ingredients mainly comprise salvianolic acid A, salvianolic acid B, protocatechuic aldehyde, dihydroisotanshione Ⅰ, tanshinlactone, tanshindiol A, and miltironel I (Zhou et al., 2005; Wang et al., 2007; Chen et al., 2014; Zhao et al., 2017) (Table 1). Previous studies have demonstrated that SM and its active ingredients possess anti-inflammatory, anti-oxidative, microcirculation-enhancing, and thrombosis-preventing effects (Cai et al., 2017; Liu C. D. et al., 2021; Liu Q. Y. et al., 2021). Tanshinone and cryptotanshinone have shown significant antibacterial effects (Li et al., 2023); while tanshinone IIA sulfonate and tanshinone have beneficial effects on the cardiovascular system. Tanshinone IIA sulfonate has been found to increase coronary blood flow, improve myocardial blood supply, alleviate the hypoxia and ischemia of myocardial tissue, and provide antithrombotic effect (Zhu et al., 2023). Additionally, tanshinol can dilate coronary artery and effectively improve the hypoxia and ischemia of myocardial tissue, and enhance ventricular diastolic function (Yin et al., 2017) The ingredients of SM demonstrate promising therapeutic effects in the treatment of cardiovascular diseases. Numerous formulations made from SM extracts have been approved as commercial medicines in China, including Danshen injection, tanshinone IIA sodium sulfonate injection, salvia polyphenolic acid salt injection, compound Danshen tablets, and compound Danshen dripping pills, which are used for the treatment of coronary heart disease, angina pectoris, myocardial infarction, and other related conditions. Additionally, Danshensu sodium injection and salvianolic acid B injection are being evaluated in clinical trials for the treatment of angina pectoris of coronary heart disease (Table 2).

**TABLE 1:** Chemical structure of main bioactive ingredients of *Salvia miltiorrhiza*.

Number	Name	Molecular formula	Chemical construction
1	Tanshinol	C_9_H_10_O_5_	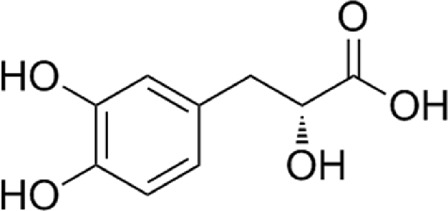
2	Salvianolic acid B	C_36_H_30_O_16_	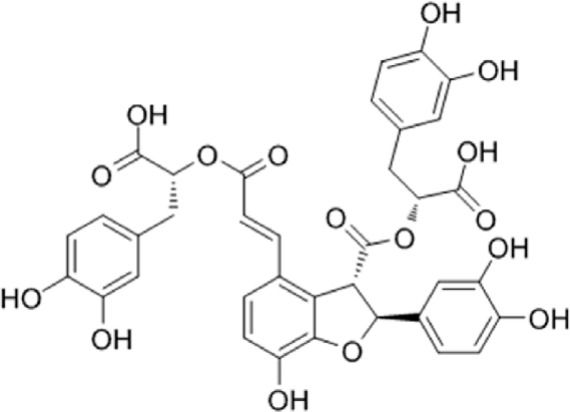
3	Protocatechuic aldehyde	C_7_H_6_O_3_	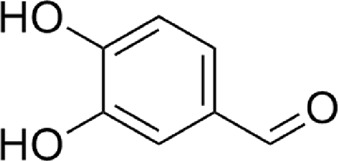
4	Tanshinone ⅡA	C_19_H_18_O_3_	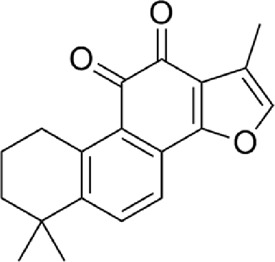
5	Cryptotanshinone	C_19_H_20_O_3_	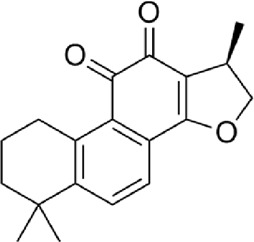
6	Tanshinone Ⅰ	C_18_H_12_O_3_	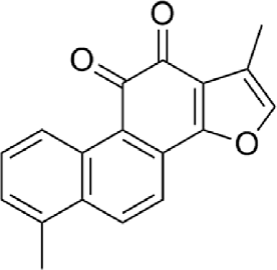

**TABLE 2:** Summary of commercial drugs of *Salvia miltiorrhiza* and its active ingredients.

Drug name	Dosage form	Ingredients	Time of approval and regulator	Treatment indications	Mechanisms
Tanshinone IIA sodium sulfonate injection	Injections	Tanshinone IIA sodium sulfonate	2002 (NMPA, China)	Used for coronary artery disease, angina pectoris, myocardial infarction, and also used for premature ventricular beats	Increase coronary flow and improve ischemic areas cardiac muscle of lateral circulation and local blood supply, improve myocardial metabolic disorders, increase myocardial resistance to hypoxia, inhibit Platelets aggregation and anti-thrombus formation, reduce the area of ischemic myocardial infarction in experimental animals, and enhance myocardial contractility at certain doses
Salvia polyphenolic acid salt for injection	Injections	Salvia polyphenolic acid salt	2011 (NMPA, China)	For stable angina pectoris in coronary artery disease, graded as grade I or II, with mild to moderate angina pectoris symptoms and TCM evidence of heart blood stasis, with symptoms of chest pain, chest tightness, palpitations	Promoting blood circulation, resolving blood stasis and clearing blood vessels
Tanshinone Capsules	Capsules	*Salvia miltiorrhiza* ethanol extract (cryptotanshinone, tanshinone I, tanshinone IIA, tanshinone IIB, methyl tanshinolate, hydroxytanshinone IIA, dihydrotanshinone I, tanshinxinkun A, tanshinxinkun B, tanshinxinkun C and other 9 ingredients)	2002 (NMPA, China)	This product is used for acne, tonsillitis, otitis externa, boils, carbuncles, Traumatic infections, burn infections, mastitis, cellulitis, osteomyelitis, etc.	It has broad-spectrum antibacterial effect, with strong antibacterial activity against *Staphylococcus aureus*, *Mycobacterium tuberculosis*, *Mycobacterium avium*, *Mycobacterium* acnes, Trichophyton rubrum, Trichophyton rubrum, and *Bacillus anthracis*
Danshen Chuanxiongzin Injection	Injections	Danshen, Chuanxiongzin Hydrochloride	2004 (NMPA, China)	For occlusive cerebrovascular diseases, such as cerebral blood supply insufficiency, cerebral thrombosis, cerebral embolism, and other ischemic cardiovascular diseases, such as chest tightness, angina pectoris, myocardial infarction, ischemic stroke, thrombo-occlusive vasculitis of coronary heart disease	Anti-platelet aggregation, dilate coronary arteries, reduce blood viscosity, accelerate the flow rate of red blood cells, improve microcirculation, and have anti-myocardial ischemia and myocardial infarction effect
Danshen Injection	Injections	*Salvia miltiorrhiza*	2002 (NMPA, China)	For coronary heart disease chest tightness, angina pectoris	Invigorate blood circulation, remove blood stasis, dilate the veins and nourish the heart
Compound Danshen Tablets	Tablets	*Salvia miltiorrhiza*, Panax notoginseng, Bingzhi	2002 (NMPA, China)	Treating chest paralysis caused by Qi stagnation and blood stasis, which is characterized by chest tightness and stabbing pain in the precordial region; coronary angina with the above symptoms	Promoting blood circulation and removing blood stasis, regulating Qi and relieving pain
Compound Danshen Drops	Pills	*Salvia miltiorrhiza*, Panax notoginseng, Bingqi	1994 (NMPA, China)	Treating chest paralysis caused by Qi stagnation and blood stasis, which is characterized by chest tightness and stabbing pain in the precordial region; coronary angina with the above symptoms	Promoting blood circulation and removing blood stasis, regulating “Qi” and relieving pain
Danshen Oral Liquid	Oral solution	*Salvia miltiorrhiza*	2005 (NMPA, China)	For chest paralysis, chest tightness and tingling caused by Qi stagnation and blood stasis; angina pectoris in coronary heart disease with the above symptoms	Promote blood circulation, remove blood stasis, dilate the veins and nourish the heart

**FIGURE 1:**
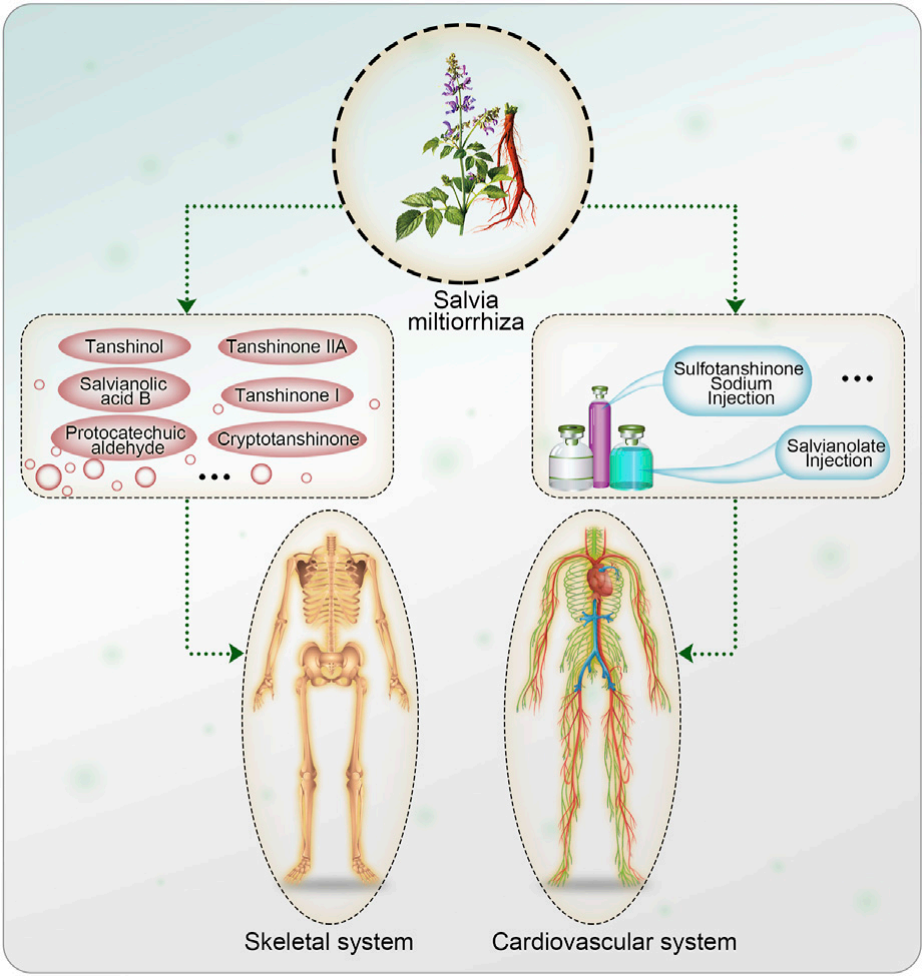
Active ingredients and formulations of *Salvia miltiorrhiza* on cardiovascular system and skeletal system.

Due to SM excellent effect of promoting angiogenesis, it is also used as an important ingredient in traditional Chinese herbal compound prescription for promoting tissue repair. In 1984, Zhang (1984) reported that SM root could enhance calcium deposition in the fracture healing process in mice, thereby promoting fracture healing. Subsequently, there has been an increasing focus on studying the protective effects of SM against MSDs, including osteoporosis, arthritis, fractures, and osteonecrosis, which pose significant health concerns. To date, active ingredients of SM such as tanshinol, salvianolic acid B, and tanshinone IIA have shown substantial protective effects on the musculoskeletal system (Figure 1). Liao Cui and other researchers (Cui et al., 2004; Luo et al., 2016; Zhang et al., 2016) found that salvianolic acid B and tanshinol induce osteogenic differentiation of bone marrow stromal stem cells (BMSCs) by upregulating the nitric oxide pathway. Additionally, they found that the water-soluble ingredients of SM had a multi-targeted effect on regulating bone metabolism and preventing osteoporosis, potentially through the regulation of oxidative stress (Yang et al., 2013). In a recent study, Gao et al. (2021) suggested that salvianolate increased bone mass in prednisone treated rheumatoid arthritis (RA) rats by modulating the RANKL/RANK/OPG signaling pathway. The skeletal protective effects of the active ingredients of SM and their therapeutic effects on skeletal diseases are increasingly being studied and extended to various musculoskeletal diseases.

**FIGURE 2:**
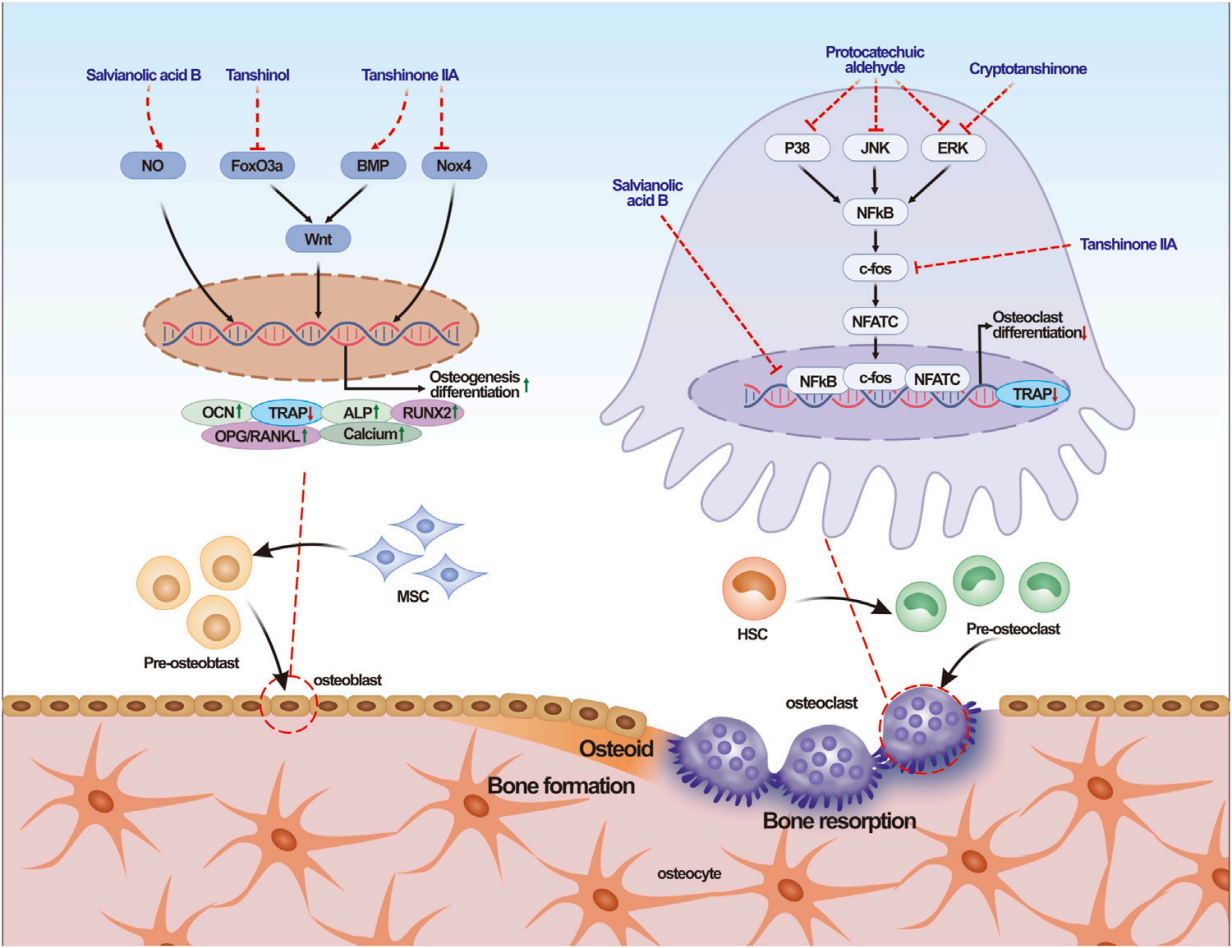
Effects and mechanism of *Salvia miltiorrhiza* and its effective ingredients on bone metabolism. Note: NO, Nitric Oxide; BMP, bone morphogenetic protein; Nox4, NADPH Oxidase 4; ALP, alkaline phosphatase; Runx2, Runt-related transcription factor 2; OCN, Ostecalcin; TRAP, Tartrate resistant acid phosphatase; RANKL, Receptor activator of nuclear factor kappa-Β ligand; OPG, Osteoprotegerin; MSC, mesenchymal stem cells; JNK, c-Jun-N-terminal-kinase; ERK, Extracellular regulated protein kinases; NF-κB, Nuclear factor kappa-B; NFATc, Nuclear Factor Of Activated T Cells; HSC, Hemapietic stem cell.

This review summarizes the effects and molecular mechanisms of SM and its active ingredients on MSD. The aim of this review is to provide evidence for the future application of SM in the prevention and treatment of MSD (Figure 2).

## 2 Effects and potential mechanism of SM and its effective ingredients on osteoporosis (OP)

### 2.1 Effects of SM and its active ingredients on osteogenesis

Osteoblasts are responsible for bone formation and play a critical role in regulating the metabolic homeostasis of bone regeneration (Abdallah et al., 2019). Previous studies have shown that the active ingredients of SM, including salvianolic acid B and tanshinone IIA, promote the proliferation and differentiation of osteoblasts, especially in glucocorticoids (GCs) induced osteoporosis (Lukert and Raisz, 1990). Salvianolic acid B has a protective effect on osteoblasts against prednisolone induced bone formation inhibition by stimulating osteoblast activity and differentiation, increasing osteogenesis marker Runx2, Osx, OCN, IGF-I, Col-I and HO-I (Qiao et al., 2019). Reactive oxygen species (ROS) has been reported to induce osteoblast apoptosis (Lee et al., 2006). MC3T3-E1 pre-osteoblasts treated with tanshinone IIA (1 µm) for 24 h reduced dexamethasone-induced apoptosis by inhibiting NADPH oxidase 4 (Nox4) expression (Li et al., 2015). Nox4 is an enzyme involved in ROS related-products (Goettsch et al., 2013). *In vivo* experiments showed that the treatment of tanshinone IIA 22 mg/kg/day in rats for 8 weeks significantly increased femoral bone mineral density, serum OCN, femoral biomachanical properties, and bone histomorphometric parameters in healthy female Wistar rats, while decreased TRAP expression (Yang et al., 2018).

Yang et al. (2013) demonstrated that salvianolic acid A (tanshinol) can attenuate oxidative stress by downregulating the FoxO3a signaling pathway and reducing oxidative stress, while upregulate the Wnt signaling pathway to counteract the inhibitory effect of H_2_O_2_ on osteogenic differentiation. Both classical and non-classical Wnt signaling pathways play important roles in bone remodeling, but the classical pathway mainly affects bone mass, while the non-classical pathway has a significant impact on bone homeostasis (Molagoda et al., 2019).

Nitric oxide (NO) is a free radical that exerts significant effects on osteoblast function. Studies have demonstrated that NO inhibits both bone resorption and bone formation, leading to reduced bone turnover in severe inflammatory conditions (van’t Hof and Ralston, 2001). Zhang X. et al. (2017) demonstrated that salvianolic acid B and tanshinol reversed the inhibitory effect of N-nitro-l-arginine methyl ester on osteogenic differentiation of bone marrow mesenchymal stem cells (BMSCs). They found that these compounds reversed the inhibition by reducing the expression of RANKL, increasing ALP, OCN, and the OPG/RANKL ratio. This modulation of the nitric oxide pathway stimulated the osteogenic differentiation of BMSCs. Oral administration of *salvia miltiorrhiza* aqueous extract 600 mg/kg/d in ovarectomized rats showed that *salvia miltiorrhiza* extract could significantly increase the bone density of trabecular bone in rats and reduce the degree of trabecular separation. In the liver of OVX rats, *salvia miltiorrhiza* could significantly reduce lipid deposition and malonaldehyde. By improving the activities of peroxidase dismutase, peroxidase, and glutathione peroxidase, *salvia miltiorrhiza* can directly improve cell proliferation and differentiation in H_2_O_2_-induced osteoblasts by reducing ROS (Dong et al., 2018).

In summary, previous studies revealed that the active ingredients of SM may reduce oxidative stress by down-regulating the FoxO3a signaling pathway while up-regulating the Wnt signaling pathway and nitric oxide pathway to promote osteoblast function and bone formation (Table 3).

**TABLE 3:** Effects and mechanism of *Salvia miltiorrhiza* and its active ingredients on osteoporosis.

Ingredient	*In vivo*/*In vitro*	Model	Administration	Dose/Time	Treatment effect	Mechanism	Diseases	References
Tanshinol	*In vitro*	Rat primary osteoblasts	-	1∼10 mg/L for 2 and 10 days	Promote ALP activity of rat cranial osteoblasts	-	OP	Cui et al. (2004)
Water extract of *Salvia miltiorrhiza*	*In vivo*; *In vitro*	Prednisone-induced osteoporosis in rats; rat primary cultured osteoblasts	Oral gavage	5 g/kg/d for 12 weeks; 10∼20 g/L for 2, 10 days	Increase the number of bone trabeculae, the weight of backbone and the content of bone organic matter. Promote ALP activity of rat cranial osteoblasts	-	OP	Cui et al. (2004)
Tanshinol	*In vivo*	Dexamethasone-treated larval zebrafish	Medicated bath	0.5∼5 μM for 3∼9 days	Reverse dexamethasone-induced inhibition of bone formation, decrease bone mass, downregulate expression of osteoblast-specific genes (Runx2, ALP, OCN, Sp7), and decrease ability to accumulate ROS production and antioxidants	-	OP	Luo et al. (2016)
Tanshinol	*In vitro*	BMSCs	-	2 × 10^−6^ M for 3, 5, 7 and 24 days	Increase the activity of ALP and the expression of OCN.	Nitric oxide pathway	OP	Zhang et al. (2017b)
Salvianolic acid B	*In vitro*	BMSCs	-	2.5 × 10^−6^ M for 3, 5, 7 and 24 days	Reverse the inhibitory effect of N-nitrol-arginine methyl ester on osteogenic differentiation of MSCs by reducing the expression of RANKL, and increase ALP, OCN and OPG/RANKL ratio	Nitric oxide pathway	OP	Zhang et al. (2017b)
Water extract of *Salvia miltiorrhiza*	*In vivo*; *In vitro*	OVX-induced osteoporosis rats; H_2_O_2_-induced preosteoblast cell	Oral gavage	600 mg/kg/d for 12 weeks	Increase bone density and reduce bone separation	-	OP	Dong et al. (2018)
Salvianolic acid B	*In vitro*	Prednisone-induced osteoblasts	-	PA 48 h and Sal B 48 h	Enhancing the activity of osteoblasts and increasing the expression of Runx2, Osx, OCN, IGF-I, Col-I and HO-I genes related to bone formation and differentiation, prednisolone acetate treatment has a protective effect on osteoblasts	-	OP	Qiao et al. (2019)
Tanshinone ⅡA	*In vitro*	Dexamethasone -induced MC3T3-E1	-	0.001∼1,000 μM for 24 h	Reverse dex-induced apoptosis	Via inactivation of Nox4	OP	Li et al. (2015)
Tanshinone ⅡA	*In vivo*	Growing rats	Oral gavage	22 mg/kg/d for 1∼2 months	Significantly increase femoral bone mineral density, maximum femoral load and bone histomorphometric parameters in healthy female wistar rats, while serum OCN levels were increase and TRAP levels were decrease in the treated rats	-	OP	Yang et al. (2018)
Tanshinol	*In vitro*	C2C12; MC3T3-E1	-	0.0001∼1,000 μM	Alleviate microcirculation disorders and bone formation disorders reversed the accumulation of ROS, decrease cell viability, cell cycle arrest, and caspase 3-dependent apoptosis caused by oxidative stress	Downregulate FoxO3a signaling and upregulate Wnt signaling under oxidative stress	OP	Yang et al. (2013)
Tanshinone ⅡA	*In vitro*	Mouse bone marrow cells and calvarial osteoblasts	-	0.5∼2.5 μg/mL for 7 days	Inhibit the osteoclast differentiation and reduce the formation of TRAP positive multinuclear osteoclasts	-	OP	Lee et al. (2005)
Tanshinone Ⅰ	*In vitro*	Mouse bone marrow cells and calvarial osteoblasts	-	0.5∼2.5 μg/mL for 7 days	Inhibit the osteoclast differentiation and reduce the formation of TRAP positive multinuclear osteoclasts	-	OP	Lee et al. (2005)
Cryptotanshinone	*In vitro*	Mouse bone marrow cells and calvarial osteoblasts	-	0.5∼2.5 μg/mL for 7 days	Inhibit the osteoclast differentiation and reduce the formation of TRAP positive multinuclear osteoclasts	-	OP	Lee et al. (2005)
Tanshinone ⅡA	*In vitro*	Osteoclasts	-	10 μg/mL for 6, 12, 24, 48 h	Inhibit osteoclast differentiation	Inhibit c-Fos and NFATc1 expression. response to RANKL.	OP	Kwak et al. (2006)
Cryptotanshinone	*In vitro*	BMMs	-	10∼80 μM for 5 days	Inhibit RANKL-induced the increase in TRAP activity in BMMs. In addition, the expressions of osteoclastogenesis-related marker proteins and NFAT activation were suppressed by cryptotanshinone treatment in BMMs	Inhibit ERK phosphorylation and NF-κB activation	OP	Wang et al. (2019b)
Protocatechuic aldehyde	*In vitro*	Raw 264.7; BMMs	-	1, 10, 20, 30, and 50 μg/mL for 5∼6 days	Reduce osteoclast formation and bone resorption, and suppress the expression of mRNA encoded proteins associated with osteoclasts	Regulate NF-κB and MAPK pathways	OP	Qu et al. (2021)
Tanshinone	*In vivo*; *In vitro*	OVX-induced osteoporosis rats; BMSCs	Tail intravenous injection	10 mg/kg/d for 2 weeks	Potently suppress OVX-induced osteoporosis and BMSC senescence	Through upregulation of PHGDH.	OP	Wang et al. (2019b)
Tanshinone ⅡA	*In vitro*	BMSCs	-	1, 5 μM for 7 and 24 days	Promote osteogenesis and inhibit osteoclastogenesis	Upregulate BMP and Wnt signaling	OP	Qian et al. (2015)
Salvianolic acid B	*In vivo*; *In vitro*	prednisone-induced osteoblasts; BMSCs	Oral gavage	40 and 80 mg/kg/d for 12 weeks; 10^−6^ mol/L∼10^−7^ mol/L for 14 days	Treat GC-induced bone loss in rats by stimulating osteogenesis, bone marrow angiogenesis and inhibiting lipogenesis	Dkk-1/β-catenin pathway	OP	Cui et al. (2012)
Tanshinol	*In vivo*; *In vitro*	prednisone-induced osteoporosis in rats; EA.hy926; MG63	Oral gavage	25 mg/kg/d for 14 weeks	Reduce microcirculation and bone formation disorders	Downregulate Wnt and VEGF pathways	OP	Lai et al. (2021)
Salvianolic acid B	*In vivo*; *In vitro*	rats spinal fusion model; EA hy9.26	Surgical implantation	The concentrations of SB in the scaffolds were 0.26% (w/w, the mass ratio of PLGA to β-TCP to SB was 80:20:0.26), 1.3% and 2.60%, 8 weeks	Enhance bone fusion by promoting osteogenesis and angiogenesis	-	Spinal fusion	Lin et al. (2019)

Note: OP, osteoporosis; ALP, alkaline phosphatase; Runx2, Runt-related transcription factor 2; OCN, ostecalcin; Osx/Sp7, Osterix; ROS, Reactive Oxygen Species; IGF-I, insulin-like growth factor 1; Col-I, type I collagen protein; HO-I, Heme Oxygenase-1; PA, Prednisolone acetate; Nox4, NADPH Oxidase 4; TRAP, tartrate resistant acid phosphatase; BMSCs, bone mesenchymal stem cells; RANKL, Receptor activator of nuclear factor kappa-B ligand; OPG, Osteoprotegerin; NFATc1, Nuclear Factor Of Activated T Cells 1; BMMs, bone marrow-derived macrophages; ERK, Extracellular regulated protein kinases; NF-κB, Nuclear factor kappa-B; MAPK, mitogen-activated protein kinase; OVX, Ovariectomy; PHGDH, phosphoglycerate dehydrogenase; BMP, bone morphogenetic protein; GC, Glucocorticoid; DKK-1, Dickkopf-related protein; VEGF, Vascular endothelial growth factor; PLGA, poly(lactic-co-glycolic acid); β-TCP, β-Tricalcium Phosphate.

### 2.2 Effects of SM and its active ingredients on osteoclastogenesis

Osteoporosis is primarily caused by an imbalance between bone resorption and bone formation, and excessive activation of osteoclasts significantly contributes to bone loss (Kikuta and Ishii, 2018). Studies demonstrated that active ingredients of SM, such as tanshinone IIA, tanshinone I, and cryptotanshinone, can reduce the formation of TRAP positive osteoclasts and suppress bone resorption (Lee et al., 2005). Kwak HB et al. found that tanshinone IIA downregulates RANKL-induced C-Fos and NFATc1, thereby inhibiting osteoblast differentiation. In the early stage of RANK signaling, activation of NF-κB and MAPKs can induce the production of c-Fos, while NFATc1 is an important regulator of RANKL-induced osteoclastogenesis. NFATc1 expression in osteoclast precursors can be upregulated by activating transcription factors such as NF-κB and activator protein-1 (AP-1) (Grigoriadis et al., 1994; Park et al., 2017; Liu et al., 2020). Wang W demonstrated that cryptotanshinone inhibited RANKL-induced osteoclastogenesis and NFATc1 expression in bone marrow macrophages (BMM) by regulating ERK and NF-κB signaling pathways (Wang W. et al., 2019). Subsequently, in a study of RAW264.7 and BMM cells, it was found that the water-soluble active ingredient of SM, protocatechuic aldehyde, reduced osteoclast formation and bone resorption, and inhibited osteoclast differentiation by inhibiting NF-κB and MAPK signaling pathways (Qu et al., 2021). However, further investigation is needed to clarify whether cryptotanshinone binds with procatechaldehyde and other active ingredients through homologous receptors on the surface of precursor cells, or directly enters the cells and inhibits the activation of NF-κB and MAPK pathways (Table 3).

### 2.3 Effects of SM and its active ingredients on bone marrow mesenchymal stem cell differentiation

BMSCs as one of the most critical cells in the MSC lineage, possessing a robust self-renewal capacity and the ability to differentiate into various cell types including osteoblasts, chondrocytes, and adipocytes. These cells play a pivotal role in bone development and remodeling. Imbalance in BMSC differentiation towards osteogenesis and adipogenesis is implicated in the pathogenesis of osteoporosis (Pittenger et al., 1999; Corrado et al., 2017; Qu et al., 2021). Ovariectomy (OVX) is a widely used model to induce osteoporosis in adult female rats. Wang L. et al. (2019) reported that OVX can induce hypermethylation of the promoter region of glycerol phosphate dehydrogenase (PHGDH), while treatment with tanshinone effectively inhibits OVX-induced osteoporosis and senescence of BMSCs by inhibiting PHGDH methylation and upregulating PHGDH expression. Bone morphogenetic proteins (BMPs) are multifunctional cytokines belonging to the transforming growth factor-β superfamily, and the BMP signaling pathway plays a crucial role in embryonic skeletal development and postnatal bone homeostasis (Wu et al., 2016). Studies demonstrated that treatment with tanshinone IIA at concentrations of 1 and 5 μM can upregulate alkaline phosphatase (ALP) activity and calcium content during osteogenic differentiation of BMSCs. Further experiments revealed that the upregulation of ALP activity and calcium content was primarily mediated by the activation of BMP and Wnt signaling pathways. However, co-treatment with Wnt inhibitor DKK-1 or BMP inhibitor noggin significantly attenuated the osteogenic differentiation effect of tanshinone IIA, indicating that tanshinone IIA promoted osteogenic differentiation primarily through upregulation of BMP and Wnt signaling pathways (Qian et al., 2015) (Table 3).

### 2.4 Effects of SM and its active ingredients on skeletal microcirculation

Under both physiological and pathological conditions, the skeletal vasculature plays a crucial role in maintaining bone homeostasis. The bone microvasculature functions as a vital supplier of oxygen, nutrients, and cytokines to bone tissue and associated cells, driving bone formation during bone development, repair, and regeneration (Chen et al., 2020). Glucocorticoids (GC) have been known to reduced blood vessels and blood flow in bone. Among the regulators of GC-induced bone remodeling in osteoporotic bone, vascular endothelial growth factor (VEGF) is considered to be of particular importance (Pufe et al., 2003; Jiang et al., 2015). Salvianolic acid B has been found to effectively treat GC-induced bone loss in rats by stimulating osteogenesis, bone marrow angiogenesis and inhibiting adipogenesis (Cui et al., 2012). Additionally, tanshinol has shown the potential to inhibit the TXNIP signaling pathway in rats with glucocorticoid-induced osteoporosis (GIO), thereby reversing the downregulation of Wnt and VEGF signaling pathways. Consequently, this process reduces microcirculation impairment and promotes bone formation (Lai et al., 2021).

In the treatment of the rabbit ischemic necrosis of the femoral head (ANFH) model, combined therapy involving SM and BMSCs has demonstrated promising results. This combined approach promotes re-ossification and hematologic reconstruction by increasing the expression of VEGF and BMP-2 in the femoral head (Wu et al., 2019). Furthermore, in a rat spinal fusion model, the incorporation of salvianolic acid B into a PLGA/β-TCP composite scaffold has shown potential in enhancing osteointegration. The scaffold promotes osteogenesis and angiogenesis, leading to improved outcomes (Lin et al., 2019) (Table 3).

## 3 Effects and potential mechanism of SM and its active ingredients on rheumatoid arthritis

Rheumatoid arthritis (RA) is a chronic, systemic, and autoimmune disease characterized by synovial inflammation, synovial hyperplasia, and progressive destruction of cartilage and joints (Huber et al., 2006; Lefevre et al., 2009; Zhai et al., 2017). Apoptosis of synovial fibroblasts (FLS) and inflammation are important in the pathogenesis of RA (Nygaard and Firestein, 2020). Inflammatory FLS in RA, which secrete a large amount of inflammatory cytokines, exacerbate the disease progression. Abnormal proliferation and insufficient apoptosis of FLS establish a vicious cycle constituting a significant process of RA development. Previous studies have shown that protocatechualdehyde, the metabolite of 3,4-Diacetoxy benzylidene diacetate (ACP), inhibited the production of IL-1α or PMA-induced IL-1β without inhibiting total protein synthesis in synovial cells of RA patients. This may be attributed to the inhibition of the PKC signaling pathway, suggesting that protocatechualdehyde may have a restorative effect on cartilage destruction (Watanabe et al., 1993). Liu et al. (2013) found that salvinorin injection inhibited the proliferation of RA fibroblastic synovial cells (RA FLSs) by promoting apoptosis. Subsequently, it was revealed that tanshinone IIA could induce apoptosis of fibroblast-like synoviocytes in RA by blocking the G2/M phase cell cycle and mitochondrial pathway (Jie et al., 2014). Moreover, it has also been shown that tanshinone IIA promotes apoptosis of RA FLS by upregulating the expression of long-stranded non-coding RNA (lncRNA) GAS5, while enhancing the expression of cleaved caspase-3/caspase-9 and inhibiting the PI3K/AKT signaling pathway (Li et al., 2018). Liu *et al.* found that the pro-apoptotic genes B-cell lymphoma 2 (Bcl-2) associated X protein (Bax) and Fas protein, were shown to be upregulated, whereas the expression levels of the anti-apoptotic gene Bcl-2 were downregulated following *salvia miltiorrhiza* treatment on RA FLSs (Liu et al., 2015).

TNF-α is a crucial cytokine implicated in bone destruction and osteoclast formation in RA, along with IL-1, IL-6, and IL-17, which are highly expressed in RA patients. TNF-α is considered as a key cytokine in the pathogenesis of RA leading to tissue damage, meanwhile it also presents a potential target for anti-RA drug development (Uttra et al., 2019). Tanshinone IIA, known for its potent antioxidant and anti-inflammatory properties, has been shown to effectively mitigate cartilage erosion and neutrophil infiltration in the ankle joint of adjuvant-induced arthritis (AIA) mice, while reducing the expression of pro-inflammatory cytokines in serum (Zhang S. et al., 2017). Tanshinone IIA inhibited the expression and release of IL-6 and TNF-α in neutrophils and promoted the apoptosis of neutrophils, suggesting its potential in improving RA by targeting neutrophils. Additionally, Tanshinone IIA sulfonate has been found to reduce the production of IL-1β, IL-6, MMP-1, and MMP-3 in TNF-α-induced RA FLS and inhibit their proliferation, migration, invasion, and inflammation by regulating the MAPK/NF-κB pathway (Wang Z. et al., 2019). Similar findings have been reported in AIA mice, where tanshinone IIA treatment significantly reduced synovial hyperplasia, inflammatory cell infiltration, and synovial tissue erosion (Du et al., 2020). Tanshinone IIA has also been found to inhibit the proliferation, migration, and invasion of RA FLS in a time- and dose-dependent manner, effectively suppressing the expression of MMP-2, MMP-3, MMP-8, MMP-9, IL-6, IL-1β, and IL-8 in TNF-α-induced RA FLS, primarily through the regulation of MAPK, AKT/mTOR, HIF-1, and NF-kB signaling pathways. Another compound, cryptotanshinone, has been shown to effectively improve inflammation and joint destruction in type II collagen-induced arthritis (CIA) rats by inhibiting the production of RANKL-induced pro-inflammatory cytokines such as IL-1, TNF-α, and IL-17 in bone marrow macrophages, reducing MMP-9 activity, and inhibiting osteoclast differentiation. Further studies have demonstrated that cryptotanshinone inhibits the degradation of NF-κB(IκB) inhibitors *in vivo* and *in vitro*, prevents lipopolysaccharide-induced nuclear translocation of NF-κB p65 in a time- and dose-dependent manner, and significantly inhibits the DNA-binding activity of NF-κB and NF-κB-dependent luciferase activity (Wang et al., 2015).

In a CIA rat model, salvianolic acid B has been shown to attenuate oxidative stress and inflammatory responses (Xia et al., 2018). In MH7A rheumatoid arthritis fibroblasts cell line, 10 μM salvianolic acid B protects and reverses damage induced by lipopolysaccharide (LPS) by inhibiting the expression of p53 and p21, suppressing cell apoptosis, and reducing the release of MCP-1, IL-6, and TNF-α. Salvianolic acid B has also been found to upregulate miR-142-3p expression and further suppress NF-κB and JNK pathways, suggesting its anti-RA effect may mediate by miR-142-3p (Meng et al., 2019).

In conclusion, the active ingredients of SM provide beneficial effects in RA mainly due to promoting RA FLS apoptosis and inhibiting inflammation. The potential mechanism may attribute to the regulation of MAPK, and NF-κB signaling pathways. There are some reports focus on the role and mechanism of SM in secondary osteoporosis induced by RA, which is worthy of further investigation (Table 4).

**TABLE 4:** Effects and mechanism of *Salvia miltiorrhiza* and its active ingredients on rheumatoid arthritis.

Ingredient	*In vivo*/*In vitro*	Model	Administration	Dose/Time	Treatment effect	Mechanism	Diseases	References
Salvianolate	*In vivo*; *In vitro*	Prednisone-treated rheumatoid arthritis rats; TNF-α-induced MC3T3-E1	Intraperitoneal injection	20 mg/kg/d for 90 days; 1 μM for 72 h	Increase BMD and trabecular/cortical bone mass, suppress inflammation, and improve bone biomechanical properties compared to CIA control and PDN treatment; Increase Osterix, OPN and Runx2 in TNF-α-induced MC3T3-E1	Regulate RANKL/RANK/OPG Signaling	RA	Gao et al. (2021)
Protocatechuic aldehyde	*In vitro*	Human monocytes	-	10∼100 μM for 48 h/s	Inhibit IL-1α or PMA-induced IL-1β production without inhibiting total protein synthesis and without cytotoxicity	Inhibit PKC signaling pathway	RA	Watanabe et al. (1993)
*Salvia miltiorrhiza* injection	*In vitro*	RA FLSs	-	0.4 mg/mL for 24 h	Promote the apoptosis and inhibit the proliferation of RA FLSs cultured in serum	-	RA	Liu et al. (2013)
Tanshinone ⅡA	*In vitro*	RA FLSs	-	2.5∼80 μM for 24, 48, 72 h	Block the cell cycle in the G2/M phase, and regulate the protein expression of Bcl-2, Bax, and Apaf-1, the release of mitochondrial Cyt-c, and the activation of caspase-9 and caspase-3	Through blockade of the cell cycle in the G2/M phase and a mitochondrial pathway	RA	Jie et al. (2014)
Tanshinone ⅡA	*In vitro*	RA FLSs	-	1, 5,10, 20, 40 and 80 μM for 24, 48, 72 h	Reduce the activity and promote the apoptosis of RAFLS. Upregulate the expression of cleaved caspase-3/caspase-9	Upregulate lncRNA GAS5 and inhibit PI3K/AKT signaling	RA	Li et al. (2018)
*Salvia miltiorrhiza* injection	*In vitro*	RA FLS	-	0.195 and 0.39 mg/mL for 24 h	Promote the expression of apoptotic genes	-	RA	Liu et al. (2015)
Tanshinone ⅡA	*In vivo*	Adjuvant-induced arthritis mice	Intraperitoneal injection	30 mg/kg/d for 40 days	Inhibit the expression of IL-6 and TNF-α and the release of neutrophils, and promote the apoptosis of neutrophils	-	RA	Zhang et al. (2017a)
Tanshinone ⅡA	*In vivo*; *In vitro*	Collagen-induced arthritis mice; RA FLSs	Oral gavage	5 mg/kg/d for 27 days; 1, 3, and 10 μM for 24 h	Decrease the production of IL-1β, IL-6, MMP-1, and MMP-3 in TNF-α-treated RA-HFLSs, alleviate rheumatoid arthritis progression and prevent inflammation damage in joint tissues of collagen-induced arthritis mice	Block MAPK/NF-κB pathways	RA	Wang et al. (2019c)
Tanshinone ⅡA	*In vivo*; *In vitro*	Collagen-induced arthritis mice; RA FLSs	Intraperitoneal injection	30 mg/kg/d for 29 days; 5, 10, and 20 μM for 24 h	Effectively suppress the increase in mRNA expression of some matrix metalloproteinases and pro-inflammatory factors induced by TNF-α in RA-FLSs, resulting in inflammatory reactivity inhibition and blocking the destruction of the knee joint	Regulate the MAPK, AKT/mTOR, HIF-1 and NF-κB pathways	RA	Du et al. (2020)
Cryptotanshinone	*In vivo*; *In vitro*	collagen-induced arthritis in rats; Raw 264.7	oral	6, 18 mg/kg/d for 16 days; 3, 10, and 30 mM for 5 h	Effectively improve inflammation and joint destruction in CIA rats, inhibit the production of RANKL-induced pro-inflammatory cytokines such as IL-1, TNF-α and IL-17 in bone marrow macrophages, decrease the activity of MMP-9, and inhibit osteoclast differentiation	Downregulate NF-κB pathway	RA	Wang et al. (2015)
Salvianolic acid B	*In vivo*	collagen-induced arthritis in rats	oral	20 and 40 mg/kg/d for 28 days	Reduce oxidative stress and inflammation in central nervous system rats	Downregulate NF-κB pathway	RA	Xia et al. (2018)
Salvianolic acid B	*In vitro*	LPS-induced MH7A	-	10 μM for 48 h	Improve the damage of MH7A cells by LPS, increase cell vitality, inhibit apoptosis, inhibit the expression of p53 and p21, and reduce the release of MCP-1, IL-6 and TNF-α	Upregulate miR-142-3p and regulate NF-κB and JNK pathways	RA	Meng et al. (2019)

Note: RA, rheumatoid arthritis; TNF-α, tumor necrosis factor-α; CIA, Collagen Induced Arthritis; IL-1, Interleukin-1; IL-17, Interleukin-17; MMP-9, Matrix metallopeptidase 9; LPS, Lipopolysaccharide; PDN, Prednisone; RA FLSs, Rheumatoid arthritis fibroblastic synovial cells; BMD, bone mineral density; OPN, Osteopontin; PKC, protein kinase C; Bax, BCL2-Associated X; bcl-2, B-cell lymphoma-2; Apaf-1, Apoptotic Protease Activating Factor 1; Cyt-c, Cytochrome c; GAS5, Growth Arrest-specific Transcripts; NF-κB, Nuclear factor kappa-B; MAPK, mitogen-activated protein kinase; PI3K, Phosphatidylinositol-3-kinase; AKT, protein kinase B; IL-6, Interleukin-6; MMP-1, Matrix metallopeptidase 1; MMP-3, Matrix metallopeptidase 3; mTOR, mammalian target of rapamycin; HIF-1, Hypoxia inducible factor-1; MCP-1, Monocyte Chemoattractant Protein-1; JNK, c-Jun N-terminal kinase.

## 4 Effects and potential mechanism of SM and its active ingredients on osteoarthritis (OA)

Osteoarthritis (OA) is one of the most common forms of arthritis, affecting over 25% of the global population over the age of 18 (Noth et al., 2008; Martel-Pelletier et al., 2016; Chen et al., 2017). Its main pathological features include degradation of articular cartilage, thickening of subchondral bone, formation of osteophytes, synovial inflammation, degradation of knee ligament and meniscus as well as hypertrophy of joint capsule (Pountos and Giannoudis, 2017). The NF-κB pathway is a common intracellular signaling pathway for multiple inflammatory factors in chondrocytes, and acts as a sensor of oxidative stress, involving in abnormal cartilage metabolism and promoting the progression of OA (Lepetsos et al., 2019). Chondrocytes have weak proliferative ability and lack the ability to renew themselves. Inflammatory processes and the ensuing apoptosis of chondrocytes are crucial in the initiation and development of OA (Wojdasiewicz et al., 2014). Jia et al. (2017) found that treatment with 0.25–0.5 mg/kg tanshinone IIA for 28 days in an OA rat model with anterior cruciate ligament resection (ACLT) and medial meniscus (MMx) resulted in significant histopathological changes in knee cartilage in the ACLT + MMx group. Tanshinone IIA at 0.5 mg/kg significantly inhibited cartilage degradation, improved Mankin score in OA model rats, and inhibited inflammatory cell accumulation and intima synovium rupture induced by ACLT + MMX in rats. Tanshinone IIA at 0.25–0.5 mg/kg dose-dependently inhibited chondrocytes apoptosis. In the ACLT + MMx group, 0.5 mg/kg of tanshinone IIA significantly inhibited the expression of matrix metalloproteinase in articular cartilage, increased the expression of tissue metalloproteinase inhibitor, and significantly decreased IL-1β, TNF-α, and iNOS inflammatory cytokines in serum. Similarly, in the study of OA rabbit model established by ACLT, it was found that after treatment with *salvia miltiorrhiza* injection, the levels of glutamate glycoside peptide (GSH) in synovial and articular cartilage of rabbits were significantly increased than those in OA group, and malondialdehyde (MDA) in synovial and articular cartilage of rabbits were significantly decreased. It is suggested that *salvia miltiorrhiza* may prevent OA joint cartilage degeneration through anti-oxidative stress (Bai and Li, 2016). Xu et al. (2018) found that *salvia miltiorrhiza* mitigated the destruction of OA cartilage by regulating JAK2/STAT3 and AKT signaling pathways. Feng et al. (2017) found that cryptotanshinone could prevent cartilage deterioration and subchondral bone thickening in OA mice. Cryptotanshinone significantly inhibited the production of NO and PGE2 as well as the expression of COX-2, iNOS, MMP-3, MMP-13, and ADAMTS-5 induced by IL-1β *in vitro*. Additionally, cryptanshinone significantly inhibited the activation of NF-κB and MAPK stimulated by IL-1β, suggesting that the therapeutic effect of cryptanshinone on OA is based on the inhibition of NF-κB and MAPK signaling pathways. LPS can significantly induce inflammatory damage in mouse chondrogenic cells (ATDC5 cells), including suppressed cell viability, enhanced apoptosis, and increased expression of pro-inflammatory factors. Tanshinone IIA protects ATDC5 cells from LPS-induced inflammatory damage by down-regulating the expression of miR-203a and inhibiting JAK/STAT and JNK pathways (Luan and Liang, 2018). According to the study of Zhou et al. (2021), tanshinone IIA can inhibit LPS-induced chondrocyte inflammation and apoptosis by regulating the expression of miR-155 and FOXO3 signaling.

In conclusion, the active ingredients of SM have been shown to alleviate the symptoms of OA by inhibiting the apoptosis of chondrocytes and down-regulating the expression of inflammatory markers. The potential underlying mechanism of this effects may relate to the inhibition of NF-κB and MAPK signaling pathways (Table 5).

**TABLE 5:** Effects and mechanism of *Salvia miltiorrhiza* and its active ingredients on osteoarthritis.

Ingredient	*In vivo*/*In vitro*	Model	Administration	Dose/Time	Treatment effect	Mechanism	Diseases	References
Tanshinone ⅡA	*In vivo*	OA rat model established by ACLT and MMx	Intraperitoneal injection	0.25∼0.5 mg/kg/d for 28 days	Suppress articular cartilage degradation through inhibition of apoptosis and expression levels of inflammatory cytokines	-	OA	Jia et al. (2017)
*Salvia miltiorrhiza* injection	*In vivo*	OA rabbit model established by ACLT	Oral gavage	3 g/kg/d for 6 weeks	Glutathione levels in synovial and articular cartilage were increased and malondialdehyde levels were decreased	-	OA	Bai and Li (2016)
*Salvia miltiorrhiza* injection	*In vivo*	OA rat model established by ACLT and MMx	Intra-articular injection	1.05 g/d for 5 weeks	Reduce the destruction of OA articular cartilage	Activate JAK2/STAT3 and AKT pathways	OA	Xu et al. (2018)
Cryptotanshinone	*In vivo*; *In vitro*	mouse OA models; OA chondrocytes	Oral gavage	10 mg/kg/d for 16 days; 5, 10 and 20 μM for 24 h	Prevent cartilage degradation and subchondral osteosclerosis in mice OA models; Significantly inhibit the IL-1β-induced NO, GE2, COX-2, iNOS, MMP-3, MMP-13, and ADAMTS-5	Inhibit both NF-κB and MAPK signaling pathways	OA	Feng et al. (2017)
Tanshinone ⅡA	*In vitro*	LPS-induced ATDC5	-	5, 10, 15 and 20 μM for 24 h	Significantly alleviated LPS-induced ATDC5 cell inflammatory injury and downregulated the expression of miR-203a	Downregulate miR-203a and suppress JAK/STAT and JNK pathways	OA	Luan and Liang (2018)
Tanshinone ⅡA	*In vitro*	Human primary chondrocytes	-	0.1, 1, 5, and 10 μM	Inhibit LPS-induced inflammation and cell apoptosis of chondrocytes	Regulate the expression of miR-155 and FOXO3	OA	Zhou et al. (2021)

Note: OA, osteoarthritis; NF-κB, Nuclear factor kappa-B; MAPK, mitogen-activated protein kinase; VEGF, Vascular endothelial growth factor; LPS, Lipopolysaccharide; IL-1β, Interleukin-1β; MMP-13, Matrix metallopeptidase 13; ACLT, Anterior cruciate ligament resection; MMx, medial meniscus; JNK, c-Jun N-terminal kinase; NO, Nitric Oxide; PGE2, Prostaglandin E2; COX-2, cyclooxygenase-2; iNOS, Inducible Nitric Oxide Synthase; MMP-3, Matrix metallopeptidase 3; MMP-13:Matrix metallopeptidase 13; ADAMTS-5, Recombinant A Disintegrin And Metalloproteinase With Thrombospondin 5; JAK, Janus Kinase; STAT, Signal transducer and activator of transcription.

## 5 Effects and potential mechanism of SM and its active ingredients on osteonecrosis of the femoral head (ON-FH)

Osteonecrosis of the femoral head (ON-FH) is a refractory and disabling hip disease caused by reduced blood supply to the femoral head and impaired bone marrow cells (Zhang et al., 2016; Mont et al., 2020). In recent years, traditional Chinese medicine demonstrated significant advantages in the treatment of femoral head necrosis. *Salvia miltiorrhiza* has been widely used in clinical non-surgical treatment of early and middle stage femoral head necrosis, with therapeutic effect that can improve ON-FH symptoms in patients and delay disease progression (Wang et al., 2010).

Salvianolic acid B has been found to have a significant anti-ischemic injury function, which is closely related to its antioxidant, free radical scavenging, and neuroprotection properties. Li and Wang (2017) found that salvianolic acid B could improve histopathological scores and inhibit osteoclast differentiation in a rat model of steroid-induced ON-FH. Salvianolic acid B also inhibited the expression of peroxisome proliferator-activated receptor gamma (PPARγ) and AP2 protein, while increasing the content of osteocalcin and alkaline phosphatase in steroid-induced ON-FH rats, suggesting that Salvianolic acid B prevented steroid-induced femoral head necrosis by inhibiting the expression of PPARγ. Subsequent studies by Xu et al. (2021) established a steroid-induced femoral head necrosis model (SIONFH) by intramuscular injection of methyl prednisone. Osteonecrosis of the femoral head was significantly alleviated after intraperitoneal injection of tanshinone I. In addition, tanshinone I increased the activity of alkaline phosphatase and the expression of osteoblast markers such as osteocalcin, type I collagen and osteopontin, while decreasing osteoclast markers, such as cathepsin K and tartrate-resistant acid phosphatase. Tanshinone I also reduced inflammation and oxidative stress, and activated the nuclear factor-erythrocyte 2-associated factor-2 (Nrf2) signaling pathway in the femoral head of ON-FH model (Table 6).

**TABLE 6:** Effects and mechanism of *Salvia miltiorrhiza* and its active ingredients on osteonecrosis of the femoral head.

Ingredient	*In vivo*/*In vitro*	Model	Administration	Dose/Time	Treatment effect	Mechanism	Diseases	References
*Salvia miltiorrhiza* injection	*In vivo*	Rabbit model of avascular necrotic femoral head	Injection	2 mL for 3 weeks and 6 weeks	Promote vascular recanalization by increasing the expression of VEGF and BMP-2 in femoral vein head	-	ONFH	Wu et al. (2019)
Salvianolic acid B	*In vivo*	Steroid-induced osteonecrosis of the femoral head in rats	Injection	40 mg/kg/d for 3 weeks	Increase the expression levels of Runx2 and Col-I	Inhibit the expression levels of PPARγ and AP2 proteins	ONFH	Li and Wang (2017)
TanshinoneⅠ	*In vivo*	Steroid-induced osteonecrosis of the femoral head rats	Intraperitoneal injection	5, 10 mg/kg/d for 4 weeks	Increase alkaline phosphatase activity and expressions of osteoblastic markers including OCN, Col-I, OPN, and Runx2 and decreased TRAP and expressions of osteoclastic markers including cathepsin K and acid phosphatase 5	Activate the Nrf2 Signaling Pathway	SIONFH	Xu et al. (2021)

Note: ON-FH, osteonecrosis of the femoral head; SIONFH, steroid-induced femoral head necrosis; VEGF, Vascular endothelial growth factor; BMP-2, bone morphogenetic protein-2; Runx2, Runt-related transcription factor 2; Col-I, type I collagen protein; OCN, Ostecalcin; OPN, Osteopontin; TRAP, Tartrate resistant acid phosphatase; (PPAR)γ, peroxisome proliferator-activated receptor; AP2, Adipocyte protein 2.

## 6 Effects and potential mechanism of SM and its active ingredients on fracture healing

Fracture is one of the most common injuries worldwide, and its high incidence imposes a significant economic burden on society (Amin et al., 2014). Clinically, a fracture that has not healed completely for more than 3 months is referred to as “delayed healing,” while the absence of any healing signs after more than 9 months, it is considered “nonunion.” Studies have shown that approximately 5%–10% of fractures result in delayed or nonunion healing (Einhorn, 1998). Fracture healing is a complex pathophysiological process, typically divided into 4 phases of inflammatory response, cartilage formation, woven bone formation, and bone remodeling (Annamalai et al., 2018; Tian et al., 2019). SM had been used as one of the bioactive ingredients in the clinical fracture treatment of traditional Chinese medicine for many years. In a study of He and Shen (2014), salvianolic acid B was found to increase the activity of ALP and the secretion of osteocalcin in a time-and dose-dependent manner, thus accelerating the early fracture healing of tibia. In Liu’s study (Liu et al., 2018), tanshinol bone-targeted liposomes were formulated using pyrophosphorylated cholesterol as a bone targeting ligand for the treatment of delayed fracture. Local application of tanshinol bone-targeted liposomes to normal mouse fracture models and glucocorticoid-induced delayed fracture healing mice models significantly improved the formation and microstructure of fracture callus, accelerated the mineralization of the callus, shortened the fracture healing duration in mice, and significantly improved the biomechanical properties of the heal bone. Subsequent study on rabbit nonunion critical size defect model revealed that tanshinol bone-targeted liposomes incorporated with collagen sponge can significantly increase the expression of type II collagen, Runx2, VEGFA and osteocalcin *in vivo*, and stimulate nonunion healing by regulating histone deacetylase 3 (HDAC3) -mediated endochondral ossification (Zhou et al., 2020) (Table 7).

**TABLE 7:** Effects and mechanism of *Salvia miltiorrhiza* and its active ingredients on fracture healing.

Ingredient	*In vivo*/*In vitro*	Model	Administration	Dose/Time	Treatment effect	Mechanism	Diseases	References
*Salvia miltiorrhiza* injection	*In vivo*	Femoral fracture in C57BL mice	Oral gavage	0.5 mL/d for 14,16, 18, 22, 26, 34, 65 days	Accelerate calcium deposition	-	Delayed fracture union	Zhang (1984)
Salvianolic acid B	*In vivo*	Rats tibia fracture model	Intraperitoneal injection	40 mg/kg/d for 3 weeks	Increase ALP activity and the secretion of osteocalcin, and accelerate early fracture healing of the tibia in rats	-	Delayed fracture union	He and Shen (2014)
Tanshinol	*In vivo*	Prednisone-induced delayed fracture union mouse model	Local injection	5 mg/kg for 18 days	Accelerate callus mineralization rate and promote fracture callus formation and microstructure	-	Delayed fracture union	Liu et al. (2018)
Tanshinol	*In vivo*; *In vitro*	Nonunion rabbits; ATDC5	Loca injection	5 mg/kg for 4 weeks; 0.1∼20 μmlo/L for 24, 48 and 72 h	Stimulate bone formation in the nonunion defect rabbit model, increase the expression of P-HDAC3, collagen II, Runx2, VEGFA, and OPN *in vivo*; accelerate endochondral ossification turnover	HDAC3-mediated endochondral ossification	Nonunion Healing	Zhou et al. (2020)

Note: ALP, alkaline phosphatase; Runx2, Runt-related transcription factor 2; VEGFA, Vascular endothelial growth factor-A; OPN, Osteopontin; P-HDAC3, phosphorylated histone deacetylase; collagen II, Type 2 collagen.

## 7 Conclusion and perspectives

**FIGURE 3:**
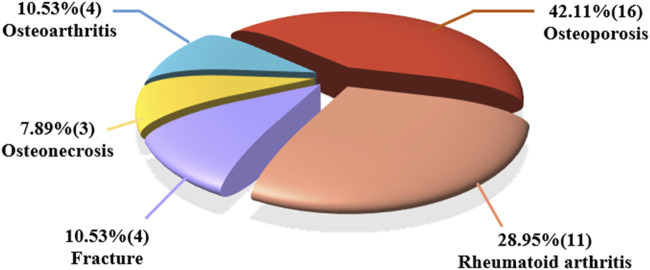
Proportion of the collected studies (38) related to *Salvia miltiorrhiza* and its active ingredients in the treatment of different types of MSD.

In this study, we conducted a comprehensive review of significant studies and demonstrated that the remarkable efficacy of SM and its active ingredients in treating MSD. Among the 38 publications collected for review, nearly half of the studies focus on osteoporosis (42.11%), followed by RA at 28.95%. Fracture repair and osteoarthritis studies account for 10.53% and 7.89%, respectively (Figure 3). It is important to highlight that the investigation of SM’s effects has expanded to cover a wide variety of MSD. Furthermore, the active ingredients found in SM show promising potential to be developed as new drugs for clinical treatment of MSD in the future.

Based on the collected publications, it appears that one of the primary mechanisms underlying the therapeutic effects of SM in treating various skeletal disorders involves the inhibition of ROS signaling. ROS signaling is implicated in a wide range of cellular processes, including apoptosis, inflammation, and cellular differentiation. By targeting ROS signaling, SM may indirectly impact multiple cellular pathways, helping to mitigate the damaging effects of oxidative stress on bone cells and tissues. Inflammatory processes, often driven by ROS, are a common feature of many musculoskeletal diseases. SM’s ability to modulate inflammatory cytokines and pathways, as discussed in the text, may be linked to its impact on ROS signaling. By reducing ROS and oxidative stress, SM may simultaneously promote osteoblast function, inhibit osteoclastogenesis, and attenuate inflammatory processes. This multifaceted approach could enhance its overall therapeutic efficacy. Further research should focus on elucidating the precise mechanisms by which SM modulates ROS signaling in different cell types and tissues. Additionally, exploring potential crosstalk between ROS-related pathways and other signaling cascades will provide a more comprehensive understanding of SM’s therapeutic effects. Understanding the central role of ROS signaling in SM’s therapeutic effects has important clinical implications.

The biggest challenge for the clinical use of SM in treating MSDs lies in the need for rigorous scientific validation through well-designed clinical trials. While preclinical studies and experimental research have shown promising results regarding the efficacy of SM and its active ingredients in various MSDs, transitioning this knowledge to clinical practice still requires robust evidence of safety and effectiveness in human subjects. Rigorous clinical trials, dose optimization, mechanistic understanding, and adherence to regulatory standards are key steps in overcoming the challenges associated with the clinical use of SM for MSDs.

## Figures and Tables

**Table:** 

**OP**	Osteoporosis
**ALP**	Alkaline phosphatase
**Runx2**	Runt-related transcription factor 2
**OCN**	Ostecalcin
**Osx/Sp7**	Osterix
**ROS**	Reactive Oxygen Species
**IGF-I**	Insulin-like growth factor 1
**Col-I**	Type I collagen protein
**HO-I**	Heme Oxygenase-1
**PA**	Prednisolone acetate
**Nox4**	NADPH Oxidase 4
**TRAP**	Tartrate resistant acid phosphatase
**BMSCs**	Bone mesenchymal stem cells
**RANKL**	Receptor activator of nuclear factor kappa-B ligand
**OPG**	Osteoprotegerin
**NFATc1**	Nuclear Factor Of Activated T Cells 1
**BMMs**	Bone marrow-derived macrophages
**ERK**	Extracellular regulated protein kinases
**NF-κB**	Nuclear factor kappa-B
**MAPK**	Mitogen-activated protein kinase
**OVX**	Ovariectomy
**PHGDH**	phosphoglycerate dehydrogenase
**BMP**	Bone morphogenetic protein
**GC**	Glucocorticoid
**DKK-1**	Dickkopf-related protein-1
**VEGF**	Vascular endothelial growth factor
**PLGA**	poly (lactic-co-glycolic acid)
**β-TCP**	β-Tricalcium Phosphate
**RA**	Rheumatoid arthritis
**TNF-α**	Tumor necrosis factor-α
**CIA**	Collagen Induced Arthritis
**IL-1**	Interleukin-1
**IL-17**	Interleukin-17
**MMP-9**	Matrix metallopeptidase 9
**LPS**	Lipopolysaccharide
**PDN**	Prednisone
**RA FLSs**	Rheumatoid arthritis fibroblastic synovial cells
**BMD**	Bone mineral density
**OPN**	Osteopontin
**PKC**	Protein kinase C
**Bax**	BCL2-Associated X
**Bcl-2**	B-cell lymphoma-2
**Apaf-1**	Apoptotic Protease Activating Factor 1
**Cyt-c**	Cytochrome c
**GAS5**	Growth Arrest-specific Transcripts 5
**PI3K**	Phosphatidylinositol-3-kinase
**AKT**	Protein kinase B
**IL-6**	Interleukin-6
**MMP-1**	Matrix metallopeptidase 1
**MMP-3**	Matrix metallopeptidase 3
**mTOR**	mammalian Target of rapamycin
**HIF-1**	Hypoxia inducible factor-1
**MCP-1**	Monocyte Chemoattractant Protein-1
**JNK**	c-Jun N-terminal kinase
**OA**	Osteoarthritis
**IL-1β**	Interleukin-1β
**MMP-13**	Matrix metallopeptidase 13
**ACLT**	Anterior cruciate ligament resection
**MMx**	Medial meniscus
**NO**	Nitric Oxide
**PGE2**	Prostaglandin E2
**COX-2**	Cyclooxygenase-2
**iNOS**	Inducible Nitric Oxide Synthase
**ADAMTS-5**	Recombinant A Disintegrin And Metalloproteinase With Thrombospondin 5
**JAK**	Janus Kinase
**STAT**	Signal transducer and activator of transcription
**ON-FH**	Osteonecrosis of the femoral head
**SIONFH**	Steroid-induced femoral head necrosis
**BMP-2**	Bone morphogenetic protein-2
**PPARγ**	Peroxisome proliferator-activated receptor gamma
**AP-2**	Adipocyte protein 2
**VEGFA**	Vascular endothelial growth factor-A
**P-HDAC3**	Phosphorylated histone deacetylase 3
**Collagen II**	Type II collagen
**MSC**	Mesenchymal stem cells
**HSC**	Hemapietic stem cell
