# Supplementary figures and images for: Expanding the therapeutic potential of Salvia miltiorrhiza: a review of its pharmacological applications in musculoskeletal diseases
Source: Front Pharmacol. 2023 Dec 5;14:1276038. doi: 10.3389/fphar.2023.1276038 (PMC10728493; doi:10.3389/fphar.2023.1276038)

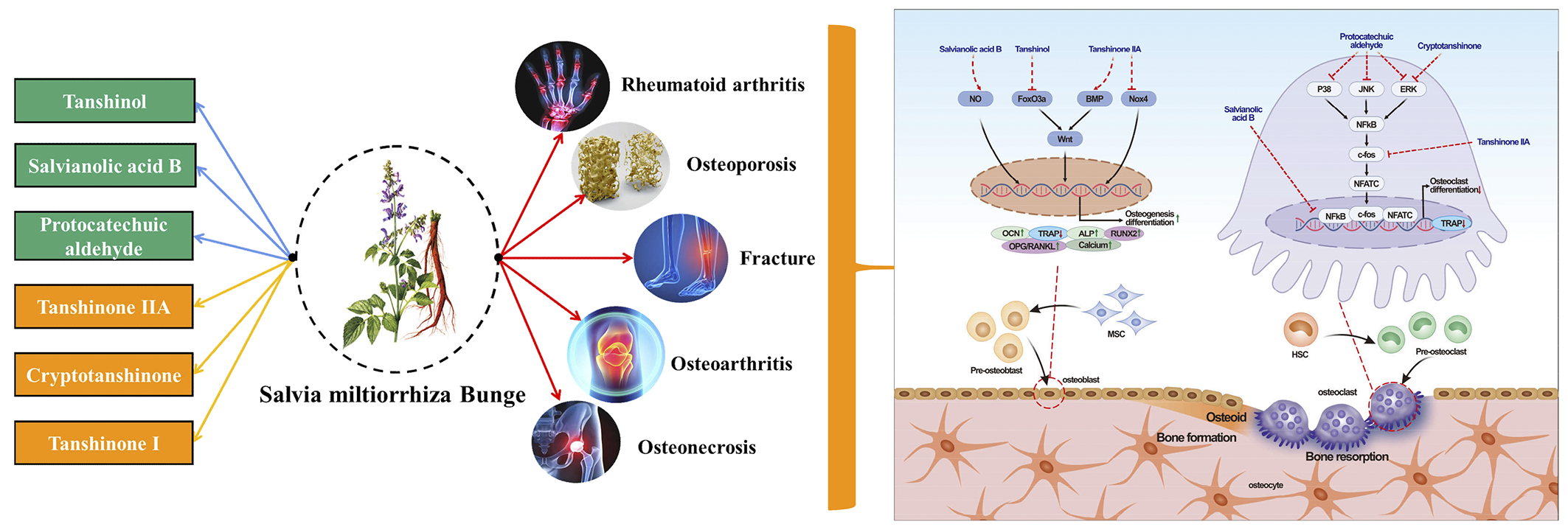

Supplement: Supplementary file 1 [file Image1.TIF]
